# Cisplatin-induced mesenchymal stromal cells-mediated mechanism contributing to decreased antitumor effect in breast cancer cells

**DOI:** 10.1186/s12964-016-0127-0

**Published:** 2016-01-12

**Authors:** Svetlana Skolekova, Miroslava Matuskova, Martin Bohac, Lenka Toro, Lucia Demkova, Jan Gursky, Lucia Kucerova

**Affiliations:** Laboratory of Molecular Oncology, Cancer Research Institute, Slovak Academy of Sciences, Vlarska 7, 833 91 Bratislava, Slovakia; Department of Plastic, Aesthetic and Reconstructive Surgery, University Hospital, Bratislava, Slovakia; Institute of Molecular and Translational Medicine, Faculty of Medicine and Dentistry, Palacky University Olomouc, Hnevotinska 5, Olomouc, Czech Republic

**Keywords:** Adipose tissue-derived mesenchymal stromal cells, Apoptosis, Cell signaling, Human breast cancer, Chemoresistance

## Abstract

**Background:**

Cells of the tumor microenvironment are recognized as important determinants of the tumor biology. The adjacent non-malignant cells can regulate drug responses of the cancer cells by secreted paracrine factors and direct interactions with tumor cells.

**Results:**

Human mesenchymal stromal cells (MSC) actively contribute to tumor microenvironment. Here we focused on their response to chemotherapy as during the treatment these cells become affected. We have shown that the secretory phenotype and behavior of mesenchymal stromal cells influenced by cisplatin differs from the naïve MSC. MSC were more resistant to the concentrations of cisplatin, which was cytotoxic for tumor cells. They did not undergo apoptosis, but a part of MSC population underwent senescence. However, MSC pretreatment with cisplatin led to changes in phosphorylation profiles of many kinases and also increased secretion of IL-6 and IL-8 cytokines. These changes in cytokine and phosphorylation profile of MSC led to increased chemoresistance and stemness of breast cancer cells.

**Conclusion:**

Taken together here we suggest that the exposure of the chemoresistant cells in the tumor microenvironment leads to substantial alterations and might lead to promotion of acquired microenvironment-mediated chemoresistance and stemness.

## Background

Breast cancer still remains a clinical challenge with considerable mortality as well as a treatment-associated morbidity. Along with a surgery and radiotherapy, the chemotherapy remains a crucial clinical modality. Better understanding of mechanisms involved in the regulation of the drug sensitivity is important for improved efficiency of cancer treatment. Recent data indicated that tumor microenvironment provides both biochemical and mechanical signaling cues to the cells and it impacts substantially on the outcome of the therapy. Direct cellular interactions and secreted paracrine factors can stimulate the tumor growth and contribute to the environment-mediated drug resistance [1–4]. There are only few studies which investigated the role of the tumor microenvironment in determining the therapeutic outcome, and, therefore, we need more experiments to predict the drug responses in patients. Pro-survival features of the stromal microenvironment may prevent efficient induction of the cell death in the tumor cells and compromise apoptotic pathways in the tumor cells embedded within the microenvironment [5, 6].

Tumor microenvironment is composed of many different types of non-malignant cells including mesenchymal stromal cells (MSC) [7]. MSC preferentially reside in perivascular niches of nearly all kinds of human tissues and display homing and engraftment potential to the injury sites in a number of pathological conditions [8]. MSC are considered cellular all-round supporters by which these cells exhibit significant sensitivity to extracellular and intracellular signals [9]. Studies by Castells et al. [5], Roodhart et al. [10] and our own data [11] indicated that MSC alter the chemosensitivity in vitro and in vivo. It was shown that carcinoma-associated mesenchymal stromal cells are able to protect ovarian cancer cells from carboplatin-induced apoptosis via inhibition of effector caspases activation and apoptosis blocade [5]. Roodhart et al. [10] have shown the production of unique fatty acids by endogenous platinum-activated mesenchymal stromal cells, that confer resistance to multiple types of chemotherapy. Moreover, Gilbert et al. [12] suggested that the chemotherapeutic drug doxorubicin leads to an acute stress response in the cells of the tumor microenvironment which results in the induction of chemoresistance in multiple myeloma. Similar processes, such as a stress response in the cells from the stromal tumor compartment including the MSC, might be involved in solid tumors as well. The MSC were shown to secrete high levels of protumorigenic cytokines IL-6 and IL-8 that might contribute to the chemoresistance and stemness, specifically if upregulated upon drug exposure [13–16]. Cancer stem cells (CSCs) can be identified and characterized using different methodologies focusing on chemoresistance, multipotency, tumorigenicity, stem cell gene expression and aldehyde dehydrogenase (ALDH) activity [17, 18].

The secretome of chemo- naïve cells and stromal compartment differs significantly from the secretion of chemotherapy-exposed cells. Although it was shown that therapy-induced secretome of tumor cells can promote resistance and tumor progression [19], recent evidence indicates that tumor-stroma coalition also play an important role in developing of drug resistance [1]. Moreover, it was reported that the MSC represented chemoresistant cells that could withstand cytotoxic stress, possessed considerable plasticity and supported tissue regeneration [20, 21]. Nevertheless, the acute secretory stress response in drug-exposed MSC and the potential effect on neighboring cells have not been examined in detail so far. Similarly to the situation in lymphatic tissues, chemotherapy might activate multiple pathways which lead to the alteration in MSC secretome and formation of resistant microenvironment in the solid tumor. MSC secrete plethora of chemokines and growth factors which were already linked to multiple regulatory functions in the tumor-associated stroma [22]. MSC affect tumor cell morphology, migratory potential and chemosensitivity [11].

In the present study we decided to examine in more detail the secretome of the drug-exposed MSC and its potential impact on the tumor cells based on the assumption that the MSC become exposed to the drugs during antitumor therapy in patients [12]. We propose that the secretory stress response might be stimulated in the MSC, as a part of tumor stroma, and thus should be considered during therapy.

## Results

### Mesenchymal stromal cells (MSC) exposed to cisplatin did not undergo apoptosis but underwent senescence

**Fig. 1:**
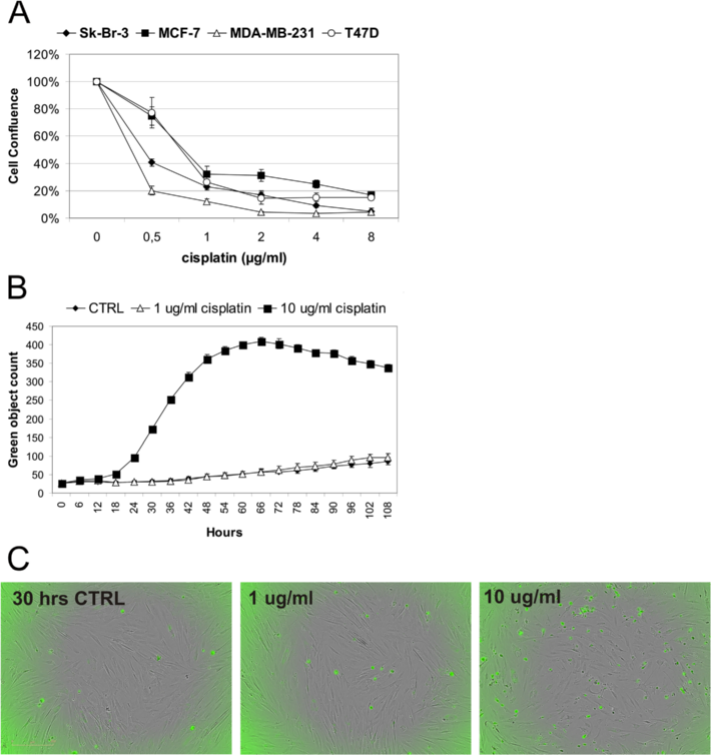
**a** MDA-MB-231, Sk-Br-3, T47D and MCF-7 tumor cells were treated with cisplatin (0.1-50 μg/ml) diluted in standard culture medium. The concentration of 1 μg/ml cisplatin (IC80 for almost all used cell lines) was set as a concentration used for pretreatment of MSC. **b** MSC were pretreated with 1 μg/ml cisplatin or 10-fold higher dose (10 μg/ml). Measurement of Caspase-3/7 activity has shown that MSC are resistant to 1 μg/ml cisplatin by corresponding to the induction of apoptosis in evaluated cells. The treatment with 1 μg/ml cisplatin did not trigger apoptosis in pretreated MSC within 48 h. MSC underwent apoptosis after more than 15 h exposure to 10 μg/ml cisplatin. **c** Using the IncuCyte Zoom™ Kinetic Imaging System we have shown that the morphology of cells treated with 1 μg/ml cisplatin remained unchanged to control and the pretreatment did not induce activation of fluorescence in cells because of missing caspase-3/7, in comparison to MSC treated with 10 μg/ml cisplatin

Based on the sensitivity of breast cancer cell lines (Fig. 1a) we have chosen the concentration of 1 μg/ml cisplatin (IC80 for almost all used cell lines) for pretreatment of MSC. To examine the sensitivity of MSC to cisplatin, we treated cells with 1 μg/ml cisplatin and 10-fold higher dose (10 μg/ml). We have shown that MSC are resistant to 1 μg/ml cisplatin by measuring Caspase-3/7 activity corresponding to the induction of apoptosis in treated cells. Treatment with 1 μg/ml cisplatin did not trigger apoptosis in MSC within 48 h. MSC underwent apoptosis after more than 15 h exposure to 10 μg/ml cisplatin (Fig. 1b). The morphology of cells treated with 1 μg/ml cisplatin remained unchanged to control, in comparison to MSC treated with 10 μg/ml cisplatin (Fig. 1c).

**Fig. 2:**
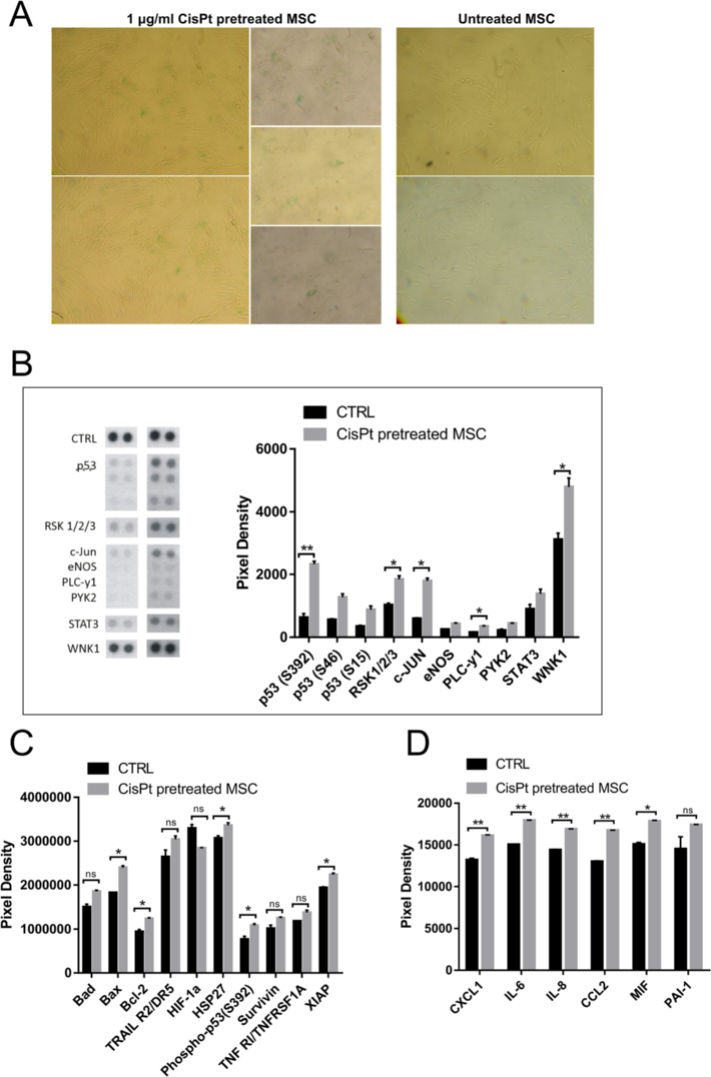
**a** Untreated and 1 μg/ml cisplatin pretreated MSC were stained to detect β-Galactosidase activity at pH 6. We have shown that part of MSC population underwent senescence after pretreatment. Cells were viewed by phase contrast in light microscope. Magnification 42x. **b** MSC were overnight pretreated with 1 μg/ml cisplatin. Analysis of phosphorylation profiles of kinases and their protein substrates revealed their increased (RSK1/2/3, WNK1, and other) or doubled (p53, c-Jun) concentration. **c** MSC overnight pretreated with 1 μg/ml cisplatin were analyzed for the relative level of apoptosis-related proteins. The pretreatment with cisplatin caused small changes in expression of both pro-apoptotic and anti-apoptotic proteins. **d** 48 h pretreatment of MSC with 1 μg/ml cisplatin increased the level of CXCL1, IL-6, IL-8, CCL2 and MIF cytokines released into MSC medium

We have observed that a part of MSC population underwent senescence after 48 h treatment with 1 μg/ml cisplatin (Fig. 2a). This phenomenon was described as the senescence-associated phenotype in mesothelioma cells describing a unique repertoire of molecules secreted by senescent cells [23]. Senescence-associated phenotype has not been described in mesenchymal stromal cells so far, as well as complete response of MSC to chemotherapy pretreatment. In order to better characterize the molecular response to cisplatin, we decided to analyse changes in signaling and secretory profile in treated MSC.

### Pre-exposed MSC activated several signaling cascades and increased secretion of several cytokines

We have analyzed the phosphorylation of different kinases and proteins in MSC and relative levels of 36 different cytokines, chemokines, and acute phase proteins in conditioned medium from the MSC pretreated with cisplatin (pr.CM) in comparison to untreated MSC (CM). The MSC were treated with 1 μg/ml cisplatin or kept in standard medium overnight, and cell lysates were prepared subsequently. We observed significantly increased phosphorylation of PLC-y1, WNK1, RSK1/2/3, p53 and c-Jun in the MSC treated with cisplatin overnight (Fig. 2b) indicating that the drug exposure activated multiple pathways but did not result in significant reduction of viability further supporting the chemoresistant properties of MSC. This observation was supported by the analysis of the apoptosis-related proteins in MSC. There were changes on both levels of the pro- and anti-apoptotic proteins; however the drug-pretreated MSC did not exhibit any indication of the pro-apoptotic signature (Fig. 2c). The analysis of conditioned medium from pretreated MSC has shown elevated levels of CXCL1, IL-6, IL-8, CCL2 and MIF cytokines in comparison to control MSC (Fig. 2d). The level of other cytokines remained unchanged or we were not able to detect them in conditioned medium.

### The effect of conditioned medium from pre-exposed MSC on breast cancer cells

**Fig. 3:**
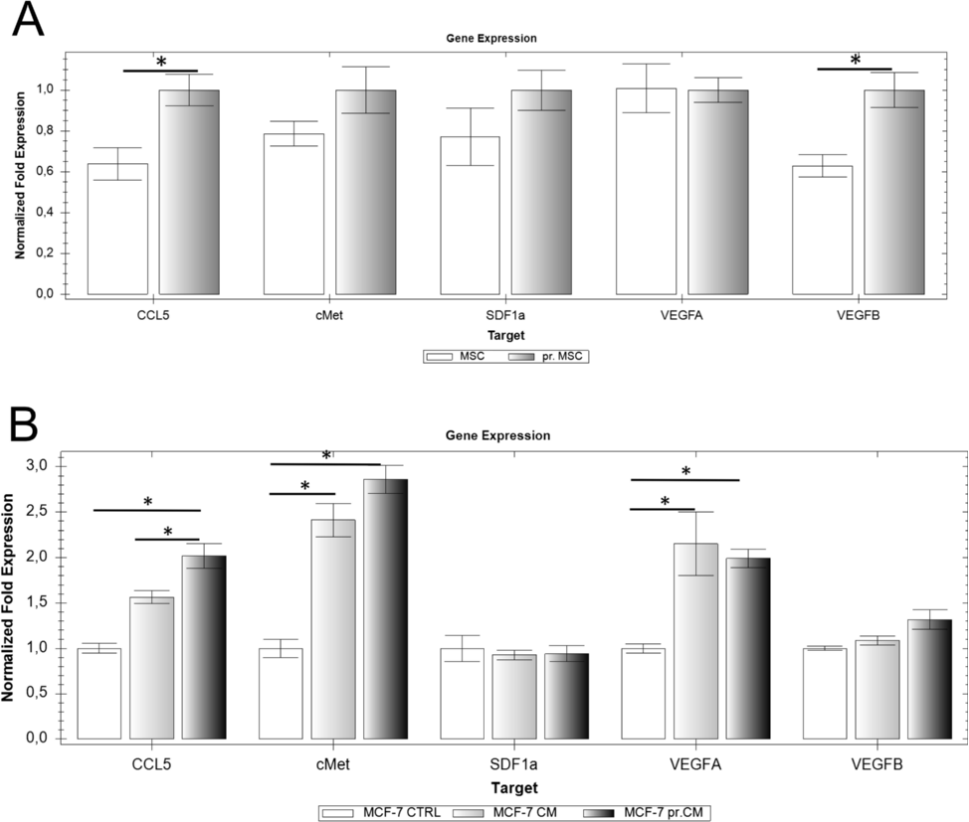
**a** Pretreatment of the MSC increased the expression CCL5, cMet, VEGFB and CXCL12 genes compared to untreated MSC (set as a control). **b** Cultivation of MCF-7 in pr.CM increased the expression of CCL5 and cMet compared to MCF-7 cultivated in CM. The expression of MCF-7 cultured under standard condition was set as a control. Gene expression was normalized to housekeeping genes β-actin and GAPDH

MSC express multiple genes responsible for invasiveness, survival, pluripotency, and mammosphere formation in breast cancer cells. Comparison of the growth factor expression profile between the drug-exposed and naïve MSC unraveled increased expression of the CCL5, cMet, VEGFB and CXCL12 in MSC pretreated with cisplatin (Fig. 3a).

Based on the substantial changes in gene expression of drug-exposed MSC, we wanted to analyze the effect of pr.CM on behavior of tumor cells in both indirect and direct co-culture. We started by the analysis of gene expression profile of tumor cells cultivated in the presence of CM vs. pr.CM. We have observed increased expression of CCL5 and cMet in tumor cells cultivated in the presence of pr.CM compared to tumor cells cultivated in CM.

**Fig. 4:**
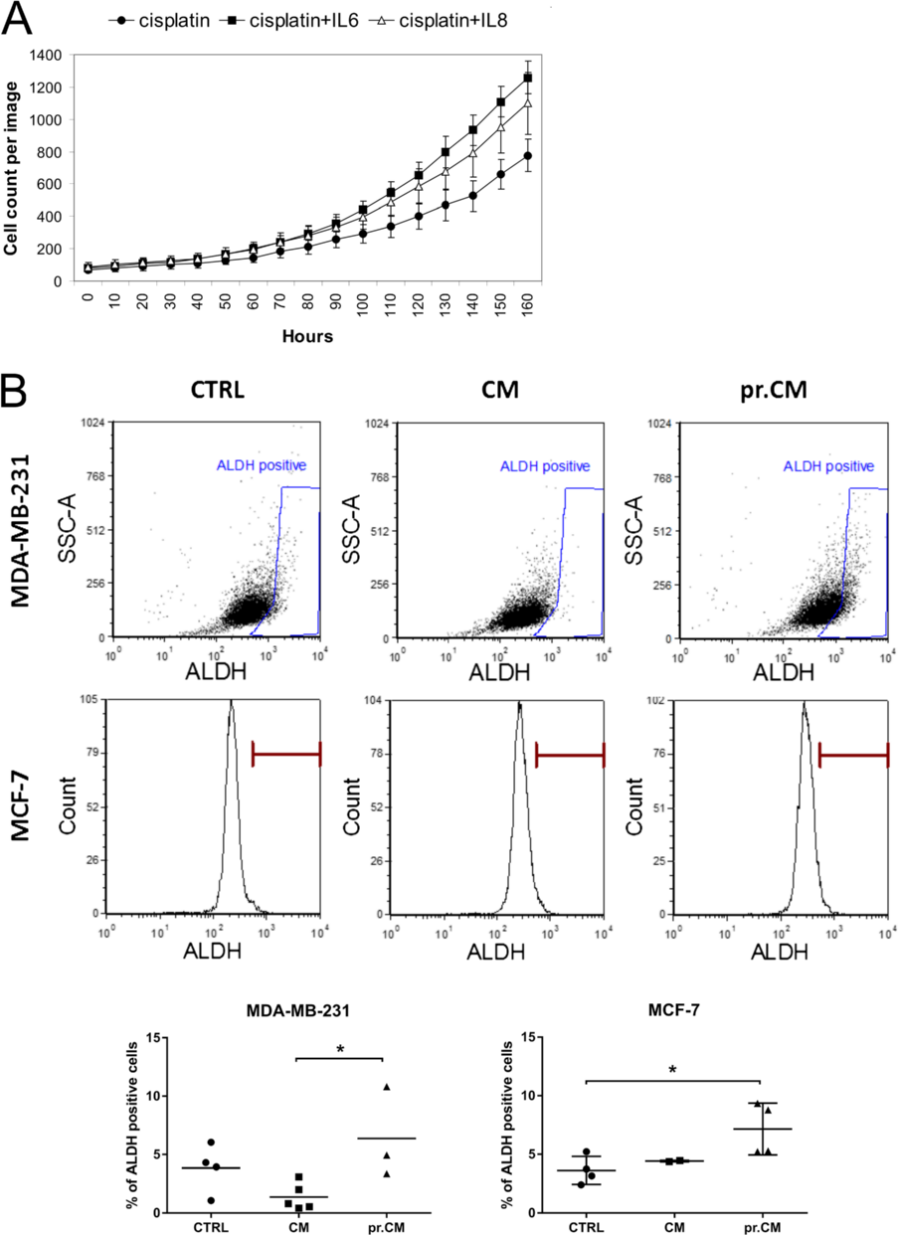
**a** MDA-MB-231 NucLight Red™ cells were treated with cisplatin (0.5 μg/ml) diluted in standard culture medium with/without 50 ng/ml IL-6, IL-8, or both. The cytokines IL-6 and IL-8 were able to increase the resistance of MDA-MB-231 NucLight Red™ cells to cisplatin. **b** Flow cytometry ALDEFLUOR® Assay has revealed increased ALDH activity in both MDA-MB-231 (10.8 % vs. 0.54 %) and MCF-7 cells (8.78 % vs. 4.46 %) cultivated in conditioned medium from pre-exposed MSC in comparison to tumor cells cultivated in control CM

We examined whether upregulated cytokines had any impact on the tumor cell sensitivity. Level of IL-6 and IL-8 cytokines, increased in pr.CM, revealed the ability to increase the resistance of MDA-MB-231 NucLight Red™ cells to cisplatin (Fig. 4a). We have also shown changes in the stemness of tumor cells cultivated in pr.CM. Conditioned medium from pre-exposed MSC increased the number of ALDH positive MDA-MB-231 cells (10.8 % in pr.CM compared to 0.54 % in CM) and MCF-7 cells (8.78 vs. 4.46 %) indicating increased population of cancer stem-like cells (Fig. 4b). We have also analyzed other stem cell-associated markers through immunostaining of CD24^−^/CD44^+^/EpCAM^+^ population in Sk-Br-3 cell line cultivated in CM or pr.CM. Data revealed increased median of fluorescence in CD24^−^/CD44^+^/EpCAM^+^ population of Sk-Br-3 cells cultivated in conditioned medium from pretreated MSC compared to cells cultivated in conditioned medium from untreated MSC (132.16 vs. 119.71) but no changes in a total number of CD24^−^/CD44^+^/EpCAM^+^ cells. It showed that cultivation in pr.CM caused an increase in the number of cell surface markers presented on the cells.

### Direct co-culture of pre-exposed MSC and tumor cells

**Fig. 5:**
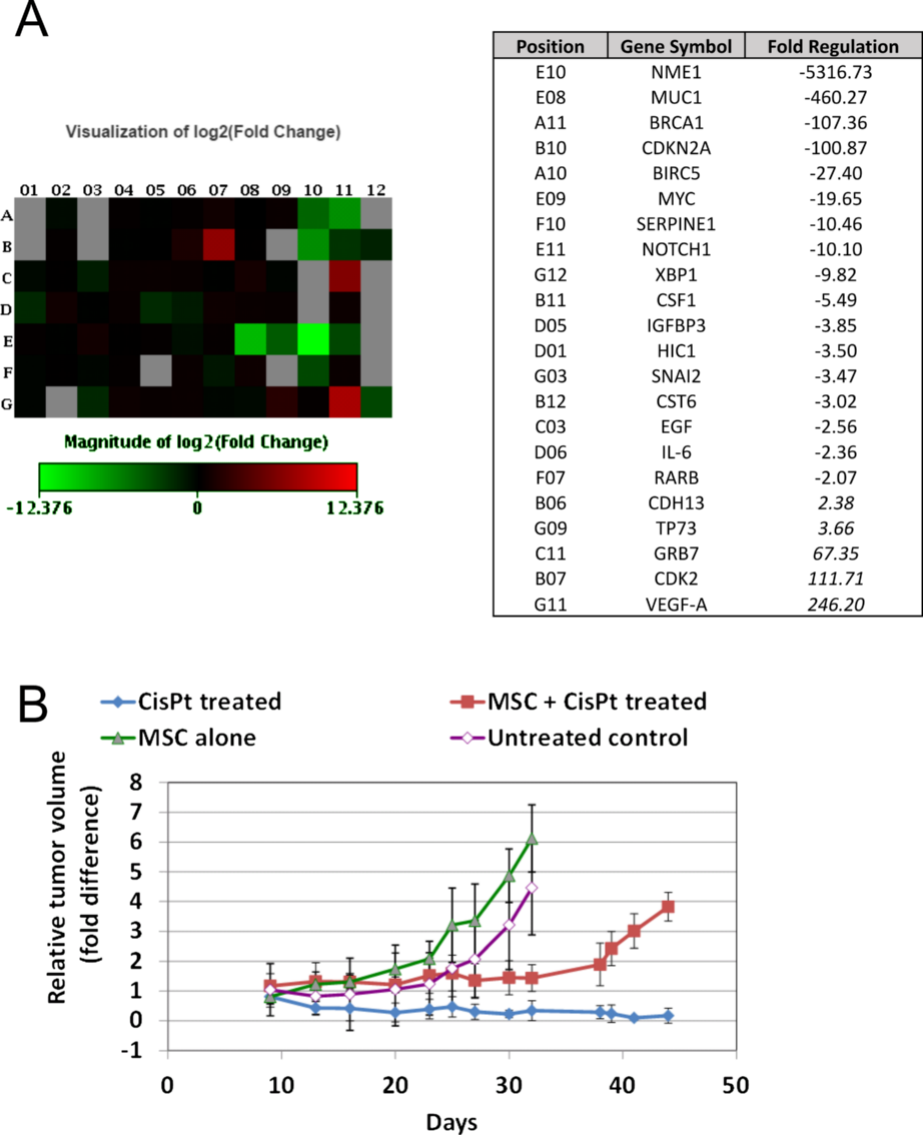
**a** MCF-7 cells and RFP-MSC (untreated or cisplatin pretretreated) were co-cultured for 5 days and subsequently sorted according to expression of fluorescent marker. Gene expression array of 84 genes revealed statistically significant downregulation or upregulation of several genes (shown in table). **b** We evaluated the effect of MSC-secreted factors also in vivo. Tumor bearing mice were treated with cisplatin (3 mg/kg) alone or in combination with 250.000 of MSC. MSC were administered *i.v*. in the same day as chemotherapy. We found that administration of MSC reduced the therapeutic effect of cisplatin

Following the analysis of indirect effect of MSC, we decided to evaluate also the effect of direct co-culture of tumor cells with untreated MSC or cisplatin pretreated MSC. MSC were retrovirally transduced with RFP, and co-cultured with tumor cells for 5 days. Subsequently, we have sorted RFP-MSC and tumor cells based on the detection of RFP. The co-culture with the same batch of MSC, which were only pretreated with cisplatin, caused significant upregulation of expression of VEGFA, CDK2, GRB7 genes, and downregulation of NME1, MUC1, BRCA1, CDKN2A, BIRC5, MYC, SERPINE1, NOTCH1 and XBP1 genes (at least 10-fold regulation) (Fig. 5a).

### Pre-exposed MSC increased the resistance of breast cancer cells in vivo

MSC alone were able to support the tumor growth of breast cancer cells in vivo in comparison to control group. The use of simultaneous treatment with cisplatin and injection of MSC led to increased resistance to cisplatin and tumor volume of MDA-MB-231 cells (Fig. 5b).

Taken together our data suggest that MSC after treatment with cisplatin are resistant to apoptosis, but activate senescence-associated phenotype, concomitantly secreting higher amounts of IL-6, IL-8, and other cytokines. This contributed to increased tumor cell chemoresistance, stemness and decreased response to chemotherapy in vivo.

## Discussion

Tumors are considered as organ-like structures than just a clonal expansion of mutant cells, and their microenvironment represents an important issue for the development of new therapeutic strategies [24, 25]. Tumor cells and their stroma are exposed to the same physiological or biological factors in the microenvironment and new studies clearly demonstrate the impact of signals derived from the cells of the tumor microenvironment on the drug response of tumor cells [26, 27].

The chemoresistance of tumor cells leading to decreased therapeutic efficiency remains one of the major obstacles in the treatment of cancer. The role of the MSC as one of the cellular component in the tumor stroma was described in both haemathological malignancies and in solid tumors [28, 29]. The MSC within the tumor microenvironment are exposed to the treatment concomitantly with the tumor cells [10] and although the chemoresistance of the MSC was described previously [15, 16], the stress response to the chemotherapy was not characterized in detail. Although we have shown that mesenchymal stromal cells are relatively resistant to chemotherapy (Fig. 1b, c), they respond to the drug exposure by several mechanisms. Our data showed that pretreatment of MSC with cisplatin stimulated secretion of different cytokines and changes in phosphorylation of many kinases (Fig. 2b-d). It resulted in increased chemoresistance and stemness of breast cancer cell lines in vitro (Fig. 3a, b) and in vivo (Fig. 5b). We have shown, that factors presented in pr.CM increase both the ALDH positivity and expression of CD24^−^/CD44^+^/EpCAM^+^ cell surface markers in tumor cells. It was demonstrated that human breast cancers contain a cell population with stem cells properties bearing the surface markers CD24^−^/CD44^+^/lin^−^ [30, 31]. Ginestier et al. [32] showed that cells bearing the overlapping phenotype of ALDH-positive and CD24^−^/CD44^+^/lin^−^ had high tumorigenic capacity and generated a tumor from as few as 20 cells.

We have characterized also the response of the MSC on chemotherapeutics and especially the effect of the soluble secreted factors released from the pretreated MSC on the chemosensitivity of the breast cancer cells. Roodhart et al. [10] showed that platinum-activated MSC secrete unique fatty acids that confer the resistance to multiple types of chemotherapy. Contrary to our data, they did not observe any effect on the tumor cells in in vitro model; and therefore suggested the requirement of secondary secreted host factors.

Castells et al. [5] showed that MSC were able to protect ovarian cells from apoptosis in response to carboplatin after stabilization of the apoptosis-inhibitory proteins. We have shown that MSC exposure to non-cytotoxic drug concentrations activated several signaling cascades. MSC pretreated with cisplatin showed increased phosphorylation of multiple tyrosine kinases such as PLC-y1, WNK1, RSK1/2/3, c-Jun, STAT3 and p53 (Fig. 2b), which could play a role in the MSC-mediated changes of tumor cells. The active form of Y705F-STAT3 was shown to drive the expression of many genes important in oncogenesis, cell cycle control and the immune response. One of these genes, CCL-5 (RANTES) was shown to be induced 42-fold by Y705F-STAT3 [33]. We have shown increased phosphorylation of Y705F-STAT3 in the MSC pretreated with cisplatin, and also increased expression of CCL5 in breast cancer cells cultivated in CM from pretreated MSC. Increased expression of CCL5 by breast tumor cells is associated with the disease progression, relapse, and metastasis; and there was reported correlation between STAT3-RANTES autocrine signaling and the acquisition of tamoxifen resistance through induction of anti-apoptotic signal, which facilitated maintenance of drug resistance [16, 34, 35]. Other influenced kinase, WNK1 was reported to be required for EGF-dependent stimulation of ERK5 without affecting the activation of ERK1/2, JNK or p38 MAP kinases [36]. Upregulated RSK family of proteins also play an important role in many biological functions, ranging from the regulation of transcription, translation and protein stability to the control of cell survival, cell motility, cell growth and proliferation [37].

We suggest that MSC in the tumor microenvironment respond to the stress mediated by chemotherapy by the secretion of cytokines and chemokines reminiscent of a senescence-associated secretory phenotype (SASP). SASP-mediated chemoresistance was described in mesothelioma cells [23] and we were able to detect β-Galactosidase activity also in cisplatin pretreated MSC (Fig. 2a) in the absence of Caspase-3/7 activation. Laberge et al. [38] showed that SASP-induced chemokines were able to influence neighboring cell population and tumor progression. Even though the senescence was partial in the MSC population, it was able to influence the level of important chemokines and cytokines that triggered changes in the exposed tumor cells. We have shown that exposure of MSC to cisplatin increased the level of CXCL1, IL-6, IL-8, CCL2 and MIF cytokines released into medium (Fig. 2d). CCL2 together with CCL5 were shown to play an important role in breast malignancy and to mediate many types of tumor-promoting cross-talks between the tumor cells and cells of the tumor microenvironment [39]. Functional analysis of tumor microenvironment revealed a correlation between CCL5 levels and IL-6 levels [40]. We have shown, that the increase of the resistance of tumor cells is partially caused also with IL-6 and IL-8 (Fig. 3a). The importance of the IL-6 and other cytokines as a prediction factor of shorter progression-free survival was shown previously in patients with ovarian cancer [41] and breast cancer (reviewed in [42]). The authors suggested the contribution of IL-6 to ascites-mediated *de novo* drug resistance. Chen et al. [43] demonstrated the role of IL-8 secreted in the MSC conditioned medium in doxorubicin resistance in the MDA-MB-231 cells. Thus the level of the IL-6 and IL-8 secretion may play an important role in the resistance mediated by the MSC exposed to chemotherapeutics.

These small changes in the level of important cytokines may play a role in expression profile, stemness and resistance of neighboring tumor cells to chemotherapy and can help tumor cells to develop complex and permanent acquired resistance.

We have analyzed also the direct co-culture of tumor cells with MSC, only pretreated with cisplatin, which changed the substantial expression of many genes compared to tumor cells cultivated with untreated MSC (Fig. 5a). We have shown increased expression of VEGFA, which play a crucial role in the stimulation of angiogenesis via signaling through VEGF receptor 2 [44], and GRB-7, which expression was shown to be strongly associated with decreased survival of breast cancer patients [45]. The most downregulated gene NME1, a well-known metastasis suppressor gene, was shown to regulate expression of genes important for distant disease-free survival and overall survival in melanoma and breast cancer [46].

We have shown that MSC alone were able to support the tumor growth and resistance of breast cancer cells also in vivo. But MSC are only one of the various cell types that constitute the tumor microenvironment and through cytokine production influence behavior of tumor cells. It was shown that also macrophages are able to promote metastatic seeding of breast cancer cells through CCL2-triggered chemokine cascade [47] or endothelial cells, which provide nutritional support to the growing tumor [48]. Andre et al. [49] discussed the predictors of chemosensitivity which could be derived from the microenvironment, but none of these markers have shown a drug specificity. They suggested a need to address the predictive value of these predictors in the context of biomarker studies.

Our experiments showed that cisplatin exposed MSC were able to produce factors that turn on the changes in stemness and resistance of tumor cells. We demonstrated that this effect is not probably caused by single specific molecule, but it is rather the result of the interplay among many cytokines concomitantly with small changes in gene expression.

## Conclusion

The tumor microenvironment is an extraordinarily heterogeneous and tumor cells are expected to experience an array of microenvironmental cues, which will in turn translate into several phenotypic manifestations. There is a great deal of evidence that points to the stroma as a major regulator of tumor progression and contributor to the risk factors determining tumor formation. It is obvious that e.g., mesenchymal stromal cells, as a part of tumor microenvironment, are exposed to therapy together with tumor cells and we can´t ignore the effects of therapy on MSC. However, they pretend to be an innocent bystanders, they smell what happens around, and after activation via treatment they can influence the tumor cells the way we haven´t expected. In conclusion, MSC were relatively resistant to cisplatin and they did not undergo apoptosis, but in contrast their secretion profile have changed, what can be important to consider when deciding the appropriate therapy for patients.

## Methods

### Cells

All chemicals were purchased from Sigma-Aldrich if not stated otherwise. Human tumor cell lines MCF-7 (ATCC® HTB-22™), Sk-Br-3 (ATCC® Number HTB-30™), T47D (ATCC® HTB-133™), MDA-MB-231 (ATCC® HTB-26™) and MDA-MB-231 NucLight Red™ (Essen BioScience, Welwyn Garden City, UK) were used for the study. Mesenchymal stromal cells (MSC) were obtained from healthy individuals undergoing elective lipoaspiration, who provided an informed consent. No humans were involved in this research study, human material harvested from the healthy individuals after elective surgery was used as approved by the Ethics Committee of the University Hospital (Ruzinov, Ruzinovska 6, 826 06 Bratislava, Slovakia). MSC were isolated and characterized by the immunophenotype and differentiation potential as previously described in [22].

Stable transduction of MSC to express red fluorescent protein (RFP) was done by retrovirus gene transfer. MSC culture was transduced three times in three consecutive days with virus containing media supplemented with 1 μg/ml protamine sulphate. Cells were maintained in selective media containing appropriate concentration of G418 for 13 days, until the control (untransduced) MSC were dead. Virus-containing medium was collected from semi-confluent cultures of GP + env-AM-12/RFP cells incubated in fresh culture medium for 24 h, filtered through 0.45 μm filter and used either fresh or kept frozen at −80 °C until use. RFP expression was confirmed by flow cytometric analysis performed on BD Canto II Cytometer (Becton Dicinson, USA).

Tumor cells were maintained in high-glucose (4.5 g/l) DMEM (PAA Laboratories GmbH, Pasching, Austria) containing 10 % FBS (GIBCO® Invitrogen, Carlsbad, CA), 10.000 IU/ml penicillin (Biotica, Part. Lupca, Slovakia), 5 μg/ml streptomycin, 2.5 μg/ml amphotericin and 2 mM glutamine (PAA Laboratories GmbH). The MSC were expanded in low glucose (1.0 g/l) DMEM supplemented with 5 % HyClone® AdvanceSTEM™ supplement (Thermo Scientific, Waltham, MA, USA) plus 5 % FBS and antibiotic/antimycotic mix (10,000 IU/ml penicillin, 5 μg/ml streptomycin and 2.5 μg/ml amphotericin) and 2 mM glutamine. Cells were maintained at 37 °C in humidified atmosphere and 5 % CO_2._

Cell-free MSC conditioned medium (CM) was collected from 2 × 10^5^ cells plated on a 35 mm culture dish after 48 h of cultivation in high-glucose medium and filtered through 0.45 μm filters. Fresh CM was always used for the experiments.

### Gene expression analysis

MSC were cultured with or without 1 μg/ml cisplatin overnight. Total RNA was isolated from 4 × 10^6^ cells. Cultured cells were collected by trypsinization, RNA isolated by NucleoSpin® RNA II kit (Macherey-Nagel, Germany) and treated with RNase-free DNase (Qiagen, Hilden, Germany). Total RNA was subjected to control PCR to confirm the absence of genomic DNA contamination. RNA was reverse transcribed with RevertAid™ H minus First Strand cDNA Synthesis Kit (Fermentas, St. Leon-Rot, Germany). 200 ng of cDNA was amplified in standard PCR performed in 8 μl 1x Dream Taq PCR Master Mix (Thermo Scientific) with 0.3 μl respective specific primers (20 pmol/μl) and DNase free water (Fermentas) in BIORAD T100™ Thermal Cycler (MJ Research, UK) with pre-set amplification profile, and horizontal electrophoresis was used for detection of amplicons.

For quantitative PCR we used following protocol: activation step at 95 °C for 3 min, 40 cycles of denaturation at 95 °C for 5 s, 10 s annealing and polymerization at 58 °C and plate read for 5 s at 75 °C followed by final extension for 5 min at 72 °C and melt curve analysis. The PCR reaction mixture (16 μl) contained 1,5 μl cDNA, 0,3 μl respective specific primers (10 pmol/μl), water and Brilliant III QPCR SYBR® Green Mix (Agilent, Santa Clara CA). qPCR reaction ran on CFX96™ Real-Time PCR Detection System (BIO-RAD Laboratories, USA).

### Drug resistance assay

For the evaluation of chemosensitivity of tumor cells, either 5 × 10^3^ Sk-Br-3, 1, 5 × 10^2^ MDA-MB-231 (resp. MDA-MB-231 NucLight Red™), 4 × 10^3^ MCF-7 or 3 × 10^3^ T47D cells were seeded in 96-well plates. On day 1, treatments were started with cisplatin (0.1–50 μg/ml) diluted in standard culture medium.

To test the effect of IL-6 and IL-8 on chemosensitivity, 1.5x10^2^ MDA-MB-231 NucLight Red™ cells were seeded in 96-well plates. On day 1, treatments were started with cisplatin (0.5 μg/ml) diluted in standard culture medium with/without 50 ng/ml IL-6, IL-8, or both.

IncuCyte Zoom™ Kinetic Imaging System and/or luminescence assay were used for analysis of treatment effects.

### Kinetic measurement of Caspase-3/7 activity

To measure caspase-3/7 activity corresponding to the induction of apoptosis in cells cultivated in the presence of cisplatin, 7.5 × 10^3^ MSC were seeded in 96-well plates and treated with 1 and 10 μg/ml cisplatin. CellPlayer 96-Well Kinetic Caspase-3/7 reagent (Essen BioScience) was used at a final concentration of 5 μM in growth medium and added directly to cells in 96-well plates. Caspase-3/7 reagent is non- fluorescent substrate which crosses the cell membrane where it is cleaved by activated caspase-3/7 resulting in the release of the DNA dye and green fluorescent staining of nuclear DNA. Kinetic activation of caspase-3/7 was monitored using IncuCyte Zoom™ Kinetic Imaging System and quantified using the IncuCyte™ FLR object counting algorithm.

### Senescence β-Galactosidase Staining

MSC were examined also for the presence of senescent cells with the Senescence β-Galactosidase Staining Kit (Cell Signaling Technology). Three × 10^5^ MSC were seeded per well in low glucose DMEM in 6-well plate, and the next day treated with/without 1 μg/ml cisplatin in standard culture medium for 48 h. The β-Galactosidase activity was detected at pH 6 by light microscopy; the blue color development indicated β-Gal-positive senescent cells.

### Flow cytometry

#### ALDH activity

ALDH activity was measured in MDA-MB-231 and MCF-7 cells cultivated in standard medium, CM or pretreated CM (pr.CM) after reaching confluence (after 4–5 days). Four hundred thousand cells were seeded on a 35 mm culture dish in standard medium, which was replaced by fresh 5 ml of standard medium, CM or pr.CM the next day. Flow cytometry ALDEFLUOR® Assay (StemCell Technologies, Vancouver, BC) was used to assess ALDH activity. Control cells were exposed to diethylaminobenzaldehyde (DEAB) prior measurement. Two hundred fifty thousand cells were centrifuged for 5 min at 250 x g, the supernatant was removed and the cells were suspended in 500 μl of ALDEFLUOR Assay buffer.

Measurement was performed using BD FACSCanto™ II Flow cytometer (Becton Dickinson, USA) equipped with FacsDiva program. Data were analysed with FCS Express program.

#### CD24^−^/CD44^+^/EpCAM^+^ activity

Sk-Br-3 cells were cultivated in standard CM or pr.CM for 5 days. CD24-PE, CD44-APC and EpCAM-FITC antibody (Miltenyi Biotec GmbH, Germany) were used at a 1:50 dilution and incubated for 15 min with 250.000 tumor cells per sample. Triple staining was used for analysis of CD24^−^/CD44^+^/EpCAM^+^ population on BD FACSCanto™ II Flow cytometer (Becton Dickinson, USA).

### Proteomic arrays

Analysis of phosphorylation profiles of kinases and their protein substrates, as well as analysis of expression of apoptosis-related proteins was done by the Human Phospho-Kinase Array (R&D Systems, Minneapolis, MN) and Human Apoptosis Array Kit (R&D Systems). For both, untreated and overnight 1 μg/ml cisplatin pretreated MSC were solubilized at 1 × 10^7^ cells/ml in lysis buffer at 2–8 °C for 30 min and proceeded according to manufacturer’s protocol. ImageJ software (NIH, Bethesda, MD) was used for the quantitative evaluation; pixel density was determined and calculated.

Cell supernatant of untreated MSC and pretreated MSC as above was analyzed by Human Cytokine Array Kit (R&D Systems) used to simultaneously detect the relative levels of 36 different cytokines, chemokines, and acute phase proteins according manufacturers protocol.

### Gene expression array

For evaluation of the effect of direct co-culture of tumor cells with MSC (untreated or pretreated with 1 μg/ml cisplatin), 200.000 of MCF-7 were cultivated with 200.000 of RFP-MSC for 5 days and then sorted on BD Influx (BD Biosciences, USA) based on RFP positivity. Excitation laser was 561 nm and emission filter 585/29. RNA from MCF-7 cells were then isolated by Agilent Total RNA Isolation Mini Kit (Agilent Technologies, USA). RNA was reverse transcribed with RT^2^ Profiler PCR Array and expression of 84 human breast cancer related genes was analyzed.

### In vivo experiments

Six week old athymic nude mice (Balb/c-nu/nu) were used in accordance with the institutional guidelines under the approved protocols. Five x10^6^ MDA-MB-231 cells were injected subcutaneously in 100 μl serum free DMEM (PAA Laboratories GmbH). Animals were subsequently divided into following groups: control group (*n* = 4), cisplatin i.p. alone (*n* = 5), i.v. 2.5 × 10^5^ MSC with i.p. cisplatin (*n* = 6), i.v. 2.5 x10^5^ MSC alone (*n* = 4). Animals were treated with 3 mg/kg cisplatin with/without MSC every 12, 19 and 26 day.

Animals were regularly inspected for the tumor growth and the tumor volume was calculated according to the formula volume = length x width^2^/2. Animals were sacrificed, when the tumors exceeded 1 cm^3^ in accordance with the ethical guidelines.

Project was performed in the approved animal facility (licence number SK PC 14011) as approved by the institutional ethic committee and by the national competence authority (State Veterinary and Food Administration of the Slovak Republic, registration number Ro 3108/14-221) in compliance with the Directive 2010/63/EU of the European Parliament and the European Council and the Regulation 377/2012 on the protection of animals used for scientific purposes.

### Statistical analysis

Studies involving comparison between the two groups were analyzed by an unpaired Student's *t*-test in GraphPad Prism® software (LA Jolla, CA). The value of p < 0.05 was considered statistically significant.

## Abbreviations

ALDH: Aldehyde dehydrogenase
CCL2: The chemokine (C-C motif) ligand 2
CM: Conditioned medium
CSCs: Cancer stem sells
CXCL1: The chemokine (C-X-C motif) ligand 1
CCL-5 (RANTES): Chemokine (C-X-C motif) ligand 5
CXCL12 (SDF-1α): C-X-C motif chemokine 12 (The stromal cell-derived factor 1 α)
DEAB: Diethylaminobenzaldehyde
ERK1/2: Extracellular signal-regulated protein kinases 1 and 2
IL-6: Interleukin-6
IL-8: Interleukin-8
MIF: Macrophage migration inhibitory factor
MSC: Mesenchymal stromal cells
Pr.CM: Pretreated mesenchymal stromal cells-medium
RSK1/2/3: Ribosomal protein S6 kinases 1–3
SASP: Senescence-associated secretory phenotype
STAT3: Signal transducer and activator of transcription 3
PAI-1: Plasminogen activator inhibitor-1
VEGF-A: Vascular endothelial growth factor A
VEGF-B: Vascular endothelial growth factor B
WNK1: WNK lysine deficient protein kinase 1
