# Erratum to: Cisplatin-induced mesenchymal stromal cells-mediated mechanism contributing to decreased antitumor effect in breast cancer cells

**DOI:** 10.1186/s12964-016-0130-5

**Published:** 2016-02-25

**Authors:** Svetlana Skolekova, Miroslava Matuskova, Martin Bohac, Lenka Toro, Erika Durinikova, Silvia Tyciakova, Lucia Demkova, Jan Gursky, Lucia Kucerova

**Affiliations:** Laboratory of Molecular Oncology, Cancer Research Institute, Slovak Academy of Sciences, Vlarska 7, 833 91 Bratislava, Slovak Republic; Department of Plastic, Aesthetic and Reconstructive Surgery, University Hospital, Bratislava, Slovakia; Institute of Molecular and Translational Medicine, Faculty of Medicine and Dentistry, Palacky University Olomouc, Hnevotinska 5, Olomouc, Czech Republic

Unfortunately, after publication of this article [1] it was noticed that 2 authors were not listed. The corrected author list can be seen above and the corrected Authors’ Contributions and Acknowledgements sections are included below.
